# Clinical Manifestations of Pulmonary Mucormycosis in Recipients of Allogeneic Hematopoietic Stem Cell Transplantation: A 21-Case Series Report and Literature Review

**DOI:** 10.1155/2022/1237125

**Published:** 2022-06-02

**Authors:** Jing Bao, Chunyu Liu, Yongxia Dong, Yu Xu, Zhanwei Wang, Kunkun Sun, Wen Xi, Keqiang Wang, Pihua Gong, Zhancheng Gao

**Affiliations:** ^1^Department of Respiratory and Critical Care Medicine, Peking University People's Hospital, Beijing 100044, China; ^2^Department of Respiratory and Critical Care Medicine, Beijing Jishuitan Hospital, Beijing 100035, China; ^3^Department of Laboratory Medicine, Peking University People's Hospital, Beijing 100044, China; ^4^Department of Pathology, Peking University People's Hospital, Beijing 100044, China

## Abstract

**Introduction:**

Mucormycosis is a rare, invasive disease caused by opportunistic pathogens related to the Mucorales order with high fatality rates in immunocompromised hosts, especially in recipients of allogeneic hematopoietic stem cell transplantation (allo-HSCT). Diagnosis and treatment of pulmonary mucormycosis in recipients of allo-HSCT remains challenging.

**Purpose:**

The aim of this study is to summarize and analyze the clinical features of pulmonary mucormycosis in recipients of allo-HSCT to explore further clinical research directions for this rare fungal infection in the particular populations.

**Methods:**

We retrospectively reviewed pulmonary mucormycosis in patients who received allo-HSCT in our hospital from January 2010 to December 2020. A total of 21 patients fulfilled the diagnostic criteria for pulmonary mucormycosis according to the European Organization for Research and Treatment of Cancer and Mycoses Study Group (EORTC/MSG) criteria. Demographic and clinical data, mycological and histopathological records, and treatment and prognosis data were collected. Clinical variables were compared between survivors and nonsurvivors. The survival days of patients with and without graft-versus-host disease (GVHD) and hemoptysis were compared separately.

**Results:**

Most of the recipients of allo-HSCT were male patients with a mean age of 43 years. Acute myeloid leukemia (AML) was the most common primary hematologic malignancy. Extrapulmonary involvement accounted for 28.6%, of the cases, including central nervous system (*n* = 5) and skin and soft tissue (*n* = 1). The median time to infection was 96 days after allo-HSCT. Clinical presentations were nonspecific, including fever (76.2%) and cough (85.7%), as well as dyspnea (19.0%), chest pain (38.1%), and hemoptysis (61.9%). Ground-glass infiltrates (95.0%) and nodules/masses (80%) were the most common radiographic patterns on chest CT. The most common pathogen was *Rhizopus* (63.2%), and breakthrough infection accounted for 90.5%. Fifteen of the patients died within one year, and the median time from diagnosis to death was 47 days.

**Conclusion:**

Mucormycosis is a fatal infection disease. Opportunistic infections in recipients of allo-HSCT are mainly breakthrough infections and may have a seasonal distribution (summer and autumn) and more cases of death in autumn. The marked reversed halo sign can be seen both in the initial stage of infection and after antifungal treatment. In our case series, patients with pulmonary mucormycosis with extrapulmonary involvement 100% died within one year. There are more patients with GVHD before infection and hemoptysis in nonsurvivors than survivors within 100 days. Patients with GVHD before infection and hemoptysis have a shorter survival time than those without.

## 1. Introduction

Mucormycosis is an invasive fungal infection caused by fungi of the order Mucorales [1]. The order Mucorales comprises numerous genera, such as *Rhizopus, Mucor, Lichtheimia, Rhizomucor, Cunninghamella,* and *Apophysomyces spp*. [2, 3]. It is widely present in environments with geographical distribution differences. By inhalation of spores, direct contact with broken wounds, and ingestion of contaminated food, this opportunistic pathogen can cause multiple system infections. Mucormycosis typically occurs in immunocompromised hosts and is less common in subjects with no underlying disease [4]. Although it is a rare disease with high mortality [5–7], increasing incidence has been noted in several centers worldwide, especially in developing countries [8].

Depending on the site of infection, mucormycosis is classified as rhinocerebral, pulmonary, cutaneous, gastrointestinal, disseminated, or other uncommon rare forms [8]. Susceptible populations include those with diabetes mellitus (DM), hematological malignancies (HMs), organ transplantation, neutropenia, autoimmune disorders, or other underlying diseases with impairments in immunity [9]. To date, few specific reports have been published about the clinical features of pulmonary mucormycosis (PM) in recipients of allogeneic hematopoietic stem cell transplantation. In this single-center retrospective study, we summarized and analyzed the clinical features of pulmonary mucormycosis in recipients of allo-HSCT to explore further clinical research directions for this rare fungal infection in this particular population.

## 2. Materials and Methods

### 2.1. Study Design

This study collected patients with pulmonary mucormycosis during the treatment of hematological malignancy in our hospital from January 2010 to December 2020. Pulmonary mucormycosis was diagnosed according to the European Organization for Research and Treatment of Cancer and Mycoses Study Group (EORTC/MSG) criteria [10]. Proven and probable infections were included in the study. Electronic hospital databases of these patients were reviewed, and demographic data, primary hematological disease and other predisposing factors, clinical presentation, radiologic features, laboratory findings, diagnostic procedures, therapeutic interventions, and clinical outcomes were collected.

Hemoptysis is defined as the expectoration of blood from the lung alveoli or airways of the lower respiratory tract [11, 12]. Extrapulmonary involvement was defined as two or more noncontiguous organ infections in patients with mucormysosis, including infection of the shin or the central nervous system, other than the respiratory system. The diagnosis time is defined as the number of days from the onset of symptoms to the receipt of a positive microbiological or histological test specimen. The survival days were defined as the time from the diagnosis of mucormycosis to the date of the last follow-up or death. The weather pattern in Beijing, China, was divided into spring, summer, autumn, and winter. Spring was considered March, April, and May; summer was considered June, July, and August; fall was considered September, October, November; and winter was considered December, January, and February. The temperature and precipitation data in Beijing were gathered from the China Meteorological Administration Government Website: https://www.cma.gov.cn.

### 2.2. Statistical Analysis

Data were analyzed using IBM SPSS Statistics version 23 and GraphPad Prism version 7.00. Fisher's exact test was used for the comparison of categorical variables, and the Mann–Whitney *U* test was used for the comparison of continuous variables. Normally distributed continuous variables are presented as the mean and standard deviation. Variables that are not normally distributed are presented as the median and interquartile range. Data are presented as frequencies and percentages for categorical variables. The x2 goodness-of-fit test was used to investigate the case distribution over the seasons. *p* < 0.05 is considered statistically significant.

### 2.3. Ethical Considerations

This study was conducted under the guidance of the Declaration of Helsinki and approved by the Ethics Committee of the Peking University of People's Hospital that waived the patients' informed consent.

## 3. Results

### 3.1. Demographic Characteristics

Twenty-one patients who received allo-HSCT were diagnosed with pulmonary mucormycosis (Table 1, Supplementary Table 1). The mean age was 43 years, and 90.5% were male. The underlying hematological diseases included acute myeloid leukemia (AML) (*n* = 12), acute lymphoblastic leukemia (ALL) (*n* = 3), myelodysplastic syndrome (MDS) (*n* = 4), chronic myeloid leukemia (*n* = 1), and aplastic anemia (*n* = 1), and all of the patients underwent allogeneic hematopoietic stem cell transplantation. Pulmonary mucormycosis-associated symptoms occurred at a median of 96 days (range: 1–1924 days) after allo-HSCT. The median time from the onset of symptoms to the receipt of a positive microbiological or histological test specimen was 19 days, the shortest diagnosis time was 4 days, and the longest was 66 days. Among the 21 patients, 8 (38.1%) were previously assessed to have graft-versus-host disease (GVHD) and were under treatment before infection. Furthermore, 4 of the 21 patients had previously experienced recurrence of hematological malignancy. Along with pulmonary mucormycosis, up to 66.7% of patients had coinfections, including bacterial and viral infections, as shown in Supplementary Table 1. Disseminated disease was observed in 6 cases (28.6%), and cerebral involvement was the most common type (*n* = 5). All patients with extrapulmonary infection died within one year, with no cases of extrapulmonary infection among surviving patients. Agranulocytosis (absolute neutrophil count <0.5 × 109/L) was present in 33.3% of the patients.

### 3.2. Clinical Manifestations

**Figure 1:**
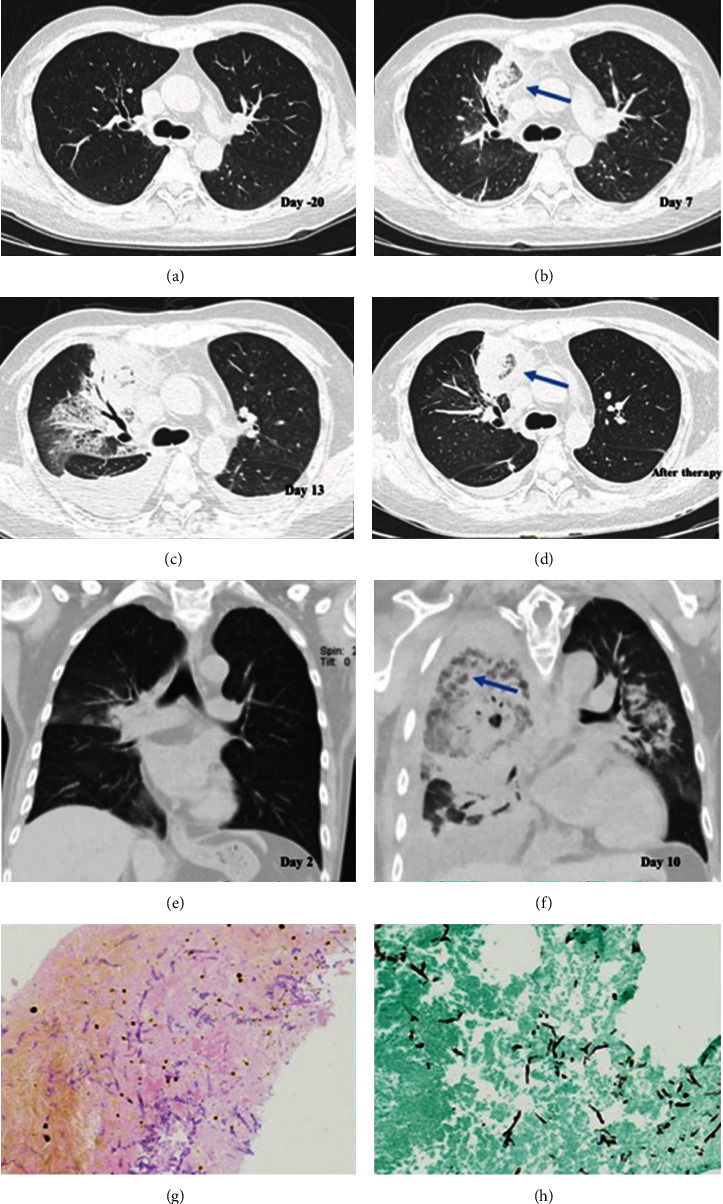
Radiographic signs and histological characteristics of PM in recipients of allo-HSCT. (a–d) The dynamic evolution of imaging during the treatment of the same patient. (a) A normal chest CT scan 20 days before infection. (b, c) Right anterior upper lobe lesions on day 7 and day 13 rapidly progressing with clinical deterioration. (d) The exudation around the lesion was significantly reduced after 3 days of application of L-AMB. A reversed halo sign was seen both in the initial stage of infection and after antifungal treatment (arrowhead). (e, f) Another patient's chest CT image. The lesion invaded the right bronchus. (g, h) Biopsy of the infected lung tissue showing ribbon-like aseptate hyphae with wide-angle branching accompanied by evidence of tissue damage under the microscope (HE stain (a), GMS stain (b), and 400x).

Clinical presentations were nonspecific, including fever (76.2%) and cough (85.7%), as well as dyspnea (19.0%), chest pain (38.1%), and hemoptysis (61.9%). Twenty patients had at least one chest computed tomography (CT) scan, and there was a wide spectrum of radiologic findings (Table 1). A total of 85.0% of infections involved multiple lobar lesions, and the most common radiologic patterns involved ground-glass infiltrates (95.0%) and nodules/masses (80.0%). Other radiologic findings included consolidation (45.0%), cavitation (50%), and pleural effusion (50.0%). Pneumothorax (5.0%) was relatively rare. Imaging signs, including the air-crescent sign and reversed halo sign, respectively, accounted for 30%. The marked reversed halo sign was observed both in the initial stage of infection and after antifungal treatment (Figure 1).

### 3.3. Microbiology and Histopathology

Eleven (52.4%) patients were diagnosed with proven pulmonary mucormycosis based on the results of histopathology and culture of sterile tissue, which was nonseptate, irregular, and ribbon-like and had Periodic Acid-Schiff (PAS) stain-positive hyphae accompanied by evidence of tissue damage under the microscope (Figure 1). At least two qualified sputum cultures were positive to diagnose probable infection for the remaining 10 patients (47.6%). Among these proven infection patients, the majority of specimens were acquired by CT-guided percutaneous lung biopsy (*n* = 6), followed by bronchoscopy (*n* = 3), surgery (*n* = 2), peripheral blood (*n* = 1), and thoracentesis (*n* = 1). One patient whose histological examination of the bronchoscopy lung biopsy specimen turned out to be negative was diagnosed with proven infection by culture. Cultures were identified at the genera level, including *Rhizopus* (63.2%), *Mucor* (31.6%), and *Rhizomucor* (5.3%).

### 3.4. Seasonal Distribution

**Figure 2:**
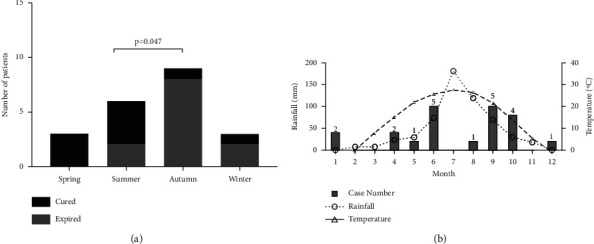
Seasonal distribution characteristics of the 21 cases. (a) Most of the cases show symptoms of infection in summer and autumn. Patients with onset in autumn had more mortality after 1 year (*p* < 0.05). (b) The temperature and rainfall in Beijing in the last decade generally peaked in July, and most of the PM patients gathered around the peak after transplantation.

The onset season of symptoms in 15 of 21 patients (71.4%) was between summer and autumn, as shown in Figure 2. There were more cases of death in autumn than in summer (*p*=0.047). The chi-square goodness-of-fit test was used to determine whether the disease had seasonal distribution characteristics for the onset time (*x*^2^ = 4.714 and *p*=0.194) and diagnosis time (*x*^2^ = 6.619 and *p*=0.085) of these 21 patients. A review of the local temperature and rainfall patterns during the last 10 years in Beijing seems to reveal an increasing number of cases with increasing temperature and rainfall, as shown in Figure 2.

### 3.5. Treatment and Outcome

**Figure 3:**
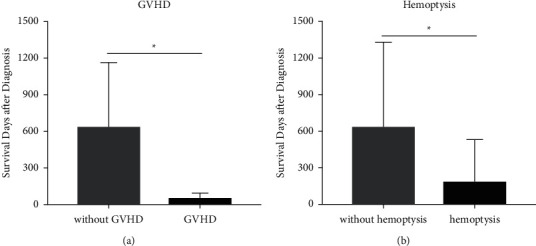
Differences of survival days between patients with or without GVHD and hemoptysis. (a) Among the 21 cases, patients with GVHD had shorter survival days than the patients without GVHD (*p*=0.043). (b) Among the 21 cases, patients with hemoptysis had shorter survival days than those who did not have hemoptysis (*p*=0.025). ^*∗*^*p* value is statistically significant.

Three (14.3%) patients did not receive targeted therapy because the diagnosis was made after death, and two (9.5%) patients accepted only once treatment for their critical clinical condition. Eighteen (78.26%) patients were treated with antifungal agents; among them, two (9.5%) patients had the opportunity to undergo surgical debridement of affected lung tissue. One patient who underwent pulmonary surgery eventually died of a brain herniation caused by central nervous system (CNS) infection. Nine (42.9%) patients accepted liposomal amphotericin B (L-AMB) in combination with posaconazole, seven (33.3%) patients accepted L-AMB monotherapy, and another 2 (9.5%) patients were treated with posaconazole monotherapy. Nineteen (90.5%) of the recipients had antifungal therapy at clinical suspicion of fungal infection before diagnosis, including fluconazole (*n* = 2), voriconazole (*n* = 10), posaconazole (*n* = 6), and echinocandins (*n* = 13). The 28-day mortality is 33.3%. The overall one-year mortality rate of patients was 71.4%, with a 100% mortality rate occurring in patients with extrapulmonary infection also called disseminated infection. The median survival time was 47 days, of which the longest was 322 days and the shortest was 1 day. Clinical variables were assessed between the survivors and nonsurvivors within 100 days after diagnosis. There was no significant difference in age, gender, history of DM, and coinfection between the two groups (Table 2). More patients with hemoptysis in nonsurvivors which accounted for 90.0% were observed (*p* < 0.05), as shown in Table 2. Moreover, patients with hemoptysis had less survival days after diagnosis (*p* < 0.05), as shown in Figure 3. It seems that more patients diagnosed with GVHD in the nonsurvivor group (*p*=0.063) in Table 2 and patients with GVHD had less survival days (*p* < 0.05) than those without. Patients died within 100 days were characterized by high C-reactive protein (CRP), low white blood cells (WBCs), multilobular involvement, and no chance for surgery but not statistically significant.

## 4. Discussion

Hematologic malignancies are one of the most common underlying diseases in mucormycosis, and pulmonary mucormycosis is the prevailing form [13]. When fungal spores are inhaled to the terminal bronchioles, the interaction of inhaled spores expressing CotH7 with the integrin *β*1 receptor of pulmonary epithelial cells and activation of the epidermal growth factor receptor (EGFR) induce fungal invasion [14]. With selective pressure from prolonged use of mold-active drugs and increasing survival among recipients of allo-HSCT patients, the incidence rate of pulmonary mucormycosis is increasing and treatment is challenging [15, 16]. Moreover, breakthrough infections reported in HSCT patients receiving *Aspergillus*-active drugs that lack anti-*Mucorales* activity are increasing [17], making it urgent to have a better understanding of pulmonary mucormycosis in recipients of allogeneic hematopoietic stem-cell transplants.

This monocentric retrospective study summarized the clinical characteristics of PM patients with allogeneic hematopoietic stem cell transplantation diagnosed by our institution in the last decade. Of all primary hematological malignancies, AML occupies the main position [18], which is consistent with our research (57.1%, 12/21). We have observed that the incidence of mucormycosis seems to be seasonally different. Up to 71.4% of the patients' initial symptoms occurred in summer and autumn. The “autumn patients” seemed to have more mortality than the “summer patients.” Shobini Sivagnanam et al. found that sinopulmonary Mucorales clusters had seasonal variation, which appeared to be related to temperature and precipitation [19]. In our study, during the last decade, the monthly average rainfall and temperature in Beijing seem to be associated with the case distribution trend. In immunocompromised patients, the main route of respiratory infection may seem to be through inhalation of sporangiospores and environmental temperature and humidity will affect the growth of Zygomycetes. A previous study demonstrated that climatic variables differentially affect airborne spore counts and invasive aspergillosis in geographically disparate centers after hematopoietic stem cell transplantation [20]. Whether invasive Mucorales infections exhibit such a seasonal pattern still needs further investigation.

Allo-HSCT is a more complicated and multifactorial process compared to autologous hematopoietic stem cell transplantation (auto-HSCT) [21]. Generally, three different phases for infection risk including (1) the early pre-engraftment phase involving 2–4 weeks after the stem cell infusion, (2) the early postengraftment phase involving 2-3 months after the HSCT, and (3) the late phase involving beyond the third month after engraftment are described in allo-HSCT. In the early pre-engraftment phase, bacteria are the leading responsible pathogens, whereas fungal pathogens are relatively less frequent. In the early postengraftment phase, GVHD-induced infection risk is increased in addition to catheter-related infections and fungal infections are frequently seen. In the late phase, the presence of chronic GVHD not only prevents immune recovery but also results in predisposition to fungal infections due to prolonged immunosuppressive treatments [21, 22]. In our study, pulmonary mucormycosis occurs in both three phases and the median time was 96 (25.5–229) days after allo-HSCT. The symptoms of PM are not specific even at late stages of infection, especially in neutropenic patients [23]. Common clinical manifestations include fever, cough, dyspnea, chest pain, and hemoptysis. Fever in our study was the most common symptom after cough, which suggests the importance of monitoring the body temperature of recipients of allogeneic hematopoietic stem-cell transplants. Especially in patients using glucocorticoids for GVHD, fever is easily ignored. One of our cases was suspected to have infection by abnormal chest CT without any symptoms. This reminds us that for recipients of allogeneic hematopoietic stem-cell transplants, the main complaint and symptoms cannot be simply used to exclude infections. Posttransplantation time, immune status, laboratory tests, and chest CT examinations can help clinicians make early diagnosis. A hallmark of mucormycosis is angioinvasion with thrombosis and tissue necrosis, which may ultimately lead to pulmonary cavitation and/or hemoptysis, which may be fatal if a major blood vessel is involved. In our case series, hemoptysis was more frequently seen in the nonsurvivor group and patients with hemoptysis had less survival days (*p* < 0.05). According to He et al.'s study [24], hemoptysis, dyspnea, and angioinvasion were independent prognostic risk factors in 12 tracheobronchial mucormycosis patients. However, in Jun Feng's study (including 12 PM patients and 80 patients published in 62 articles), hemoptysis was one of the four independent determinants associated with a better outcome [25]. The reason for this difference may be caused by the different subgroups of the patient population. The study object of this research is a special group of recipients of allogeneic hematopoietic stem-cell transplants, and it seems that hemoptysis may be an “unfriendly symptom.”

The CT features of PM usually start with perivascular ground-glass lesions [26] and then progress to consolidation, nodules, masses, and a multifocal pattern. A study showed that a multifocal pneumonia pattern was associated with a high mortality rate [26]. Limited by the means of diagnosis, it is often difficult to capture infection imaging from the very beginning. In immunocompromised patients with pulmonary mucormycosis, the most common pattern on first CT scans was nodules/masses or consolidation with surrounding ground-glass opacity halos [27]. We noticed that 95.0% (19/20) of our cases showed ground-glass infiltrates, which included a ground-glass halo with nodules/masses and simply ground-glass lesions. Both nodular or masses and consolidative patterns were seen in our cases, in which nodules or masses were more common. The hallmark CT imaging manifestation of PM is the “reversed halo sign (RHS),” which is a focal round area of ground-glass attenuation surrounded by ring consolidation (Figure 1). A photomicrograph of the edge of the lesion with the RHS shows a hemorrhagic necrotic cavity area with a transition zone containing inflammatory cells and variable fibrosis [28]. Legouge et al. demonstrated that in neutropenic leukemia patients with pulmonary infection, the presence of the RHS on CT was a strong indicator of PM [29]. The proportion of RHS in our cases was also relatively high, and the dynamic evolution of RHS can be observed on CT in many cases (Figure 1). Rarely, patients may present with an invasive endobronchial mass, which is shown in Figure 1.

A proven invasive infection requires histological analysis or culture of a sterile material or tissue nucleic acid diagnosis. The probable and possible infections were based on host factors, clinical manifestations, and mycological evidence [10]. A previous study showed that the time between the first symptoms and microbiology and/or histopathology assessment was 2 weeks [30]. According to our cases, the median time in patients who underwent allo-HSCT was 19 days. Because of the nonspecific clinical features and difficulty in distinguishing PM from other invasive fungal diseases, the frustrating long diagnosis time makes it hard for HM patients to obtain a proven diagnosis and optimal therapy is often delayed, which results in a 2-fold increase in 90-day mortality [31, 32]. Invasive procedures used to obtain sterile tissues are particularly difficult for recipients of allo-HSCT, which explains why there were only 11 proven patients of 21 patients in our cases. Because homogenization of the tissue may cause viability loss of the nonseptate, fragile hyphal forms of these fungi, half of the cases of mucormycosis are false negative [33]. In our cases, the false-negative culture was seen in 3 cases of the 11 patients. A previous study confirmed the value of quantitative polymerase chain reaction (qPCR) in BAL regarding earlier diagnosis in cases of PM [34]. Many ongoing studies focusing on noninvasive methods, such as qPCR for the detection of circulating deoxyribonucleic acid (DNA) in blood (plasma or serum) or urine, are beginning to show the value of early diagnosis and early treatment [35, 36].

Breakthrough infection is defined by prophylactic receipt of mold-active antifungal medications in mucormycosis patients. In our study, 90.5% of the PM is breakthrough infection. Selective pressure from the prolonged use of mold-active antifungal medications may increase the frequency of zygomycosis among immunosuppressed patients. It is believed that breakthrough infection on Mucorales-active antifungals portrays poor prognosis [37, 38]. Although our study did not show empirical antifungal treatment before the diagnosis of mucormycosis causing increased mortality limited by the number of cases, breakthrough infection still remains a serious issue in fungal infection after allo-HSCT. A recent study indicated that the overall prognosis in HSCT and HM populations remains extremely poor after diagnosis, with only a 37% survival rate at 1 year [39]. In our cases, the survival rate at 1 year is 28.6%. Whether the patient was experiencing GVHD before infection differed in the survivor group and the nonsurvivor group in our case series. It is believed that HSCT and immunosuppressive therapy, especially using glucocorticoids and cytotoxic drugs for GVHD treatment, might deepen and prolong the existing deficiencies in humoral and cellular immune functions [40]. It is worth noting that the surviving patients within 1 year had no extrapulmonary involvement, while the extrapulmonary involvement patients, including CNS and skin patients, had a 1-year mortality rate of 100%. Valliappan Muthu also found that disseminated disease had a higher risk of death than isolated PM [41].

The treatment of pulmonary mucormycosis requires multidisciplinary cooperation with timely diagnosis, improvement of underlying disease, and surgical removal of infected foci and antifungal therapy. The historical mainstay of antifungal therapy is amphotericin B (AmB). Limited by its substantial toxicity, the dose and treatment durations still need more advanced evidence-based support. Posaconazole (POS) with improved pharmacokinetic profiles have provided treatment options [42]. Isavuconazole (ISA) is approved as a first-line therapy if amphotericin B treatment is not appropriate [43]. Recent studies have found that a 17 kDa toxin, which plays a key role in the pathophysiological mechanism of mucormycosis, may become a promising target for the treatment of mucormycosis in the future [44]. In our study, 42.9% of the patients accepted L-AMB + POS treatment. A previous study has shown that whether patients receive monotherapy or combination treatment as initial therapy has no difference in mortality in HM patients [45]. With the improvement of molecular diagnostic technology, early diagnosis of mucormycosis is promising and the significance of combination therapy may still be discussed. Early complete surgical treatment for mucormycosis, in addition to systemic antifungal treatment, has been suggested [8, 46]. In our cases, surgical treatment accounted for 9.5% (2/21) of all HM patients. The reason for the lower surgery rate may be that primary HM causes coagulation dysfunction or multiple organ dysfunction secondary to HSCT, making surgery often impossible. There is currently no clinical research evidence for the course of antifungal treatment after surgery. One of our patients underwent surgical debridement but still died of CNS infection one year later, emphasizing the importance of maintaining antifungal therapy after surgery.

## 5. Limitations

Our study has several limitations. Firstly, this is a single-center retrospective study; thus, selection bias is inevitable. Secondly, the number of cases in this study is limited and there is a lack of appropriate disease and healthy controls. It is difficult to conduct prospective multicenter clinical trials for pulmonary mucormycosis. By summarizing the clinical manifestations of PM, we hope that this study can provide possible research directions for further clinical research in the future.

## 6. Conclusions

In this study, we presented 21 cases of pulmonary mucormycosis in recipients of allo-HSCT. This rare opportunistic infection in recipients of allo-HSCT are mainly breakthrough infections and may have a seasonal distribution (summer and autumn) and more cases of death in autumn. The marked reversed halo sign can be seen both in the initial stage of infection and after antifungal treatment. In our cases, pulmonary mocurmycosis patients after allo-HSCT with extrapulmonary involvement all died within one year. GVHD before infection and hemoptysis is more observed in nonsurvivors than survivors within 100 days. Patients with GVHD before infection and hemoptysis have a shorter survival time than those without.

## Figures and Tables

**Table 1:** Patient demographics, symptoms, radiographic findings, treatments, and outcomes.

Demographic of patients	Number of patients (%)
Age at diagnosis	43 ± 12.4
Male, *n* (%)	19 (90.5%)
Hematological malignancies, *n* (%)	
AML	12 (57.1%)
ALL	3 (14.3%)
MDS	4 (19.0%)
Others	2 (9.5%)
GVHD before infection	8 (38.1%)
Days after allo-HSCT	96 (25.5–229)
Symptom to time of diagnosis	19 (12–34)
Antifungal therapy at clinical suspicion	19 (90.5%)
Fluconazole	2 (9.5%)
Voriconazole	10 (47.6%)
Posaconazole	6 (28.6%)
Echinocandins	13 (61.9%)
Overall clinical presentation	
Multiorgan involvement, n (%)	6 (28.6%)
Agranulocytosis, *n* (%)	7 (33.3%)
Fever, *n* (%)	16 (76.2%)
Dyspnea, *n* (%)	4 (19.0%)
Cough, *n* (%)	18 (85.7%)
Chest pain, *n* (%)	8 (38.1%)
Hemoptysis, *n* (%)	13 (61.9%)
CT findings	
Nodule and/or mass	16 (80.0%)
Consolidation	9 (45.0%)
Ground-glass infiltrates	19 (95.0%)
Cavitation	10 (50.0%)
Pneumothorax	1 (5.0%)
Pleural effusion	10 (50.0%)
Imaging signs	
Air-crescent sign	6 (30.0%)
Reversed halo sign	6 (30.0%)
CT distribution	
Lobar	3 (15.0%)
Multilobar	17 (85.0%)
Origin of specimen	
Sputum, *n* (%)	10 (47.6%)
Biopsy	
CT-guided, *n* (%)	6 (28.6%)
Transbronchial lung biopsy, *n* (%)	3 (14.3%)
Pleural effusion, *n* (%)	1 (4.8%)
Peripheral blood	1 (4.8%)
Pathogen (genera)	
*Mucor*	6 (31.6%)
*Rhizopus*	12 (63.2%)
*Rhizomucor*	1 (5.3%)
Treatment and outcome of patients	
Single-agent amphotericin B, *n* (%)	7 (33.3%)
Multiagent antifungal therapy with amphotericin B, *n* (%)	9 (42.9%)
Combination medical and surgical intervention, *n* (%)	2 (9.5%)
One-year mortality, *n* (%)	15 (71.4%)
28-day mortality, *n* (%)	7 (33.3%)
Time from diagnosis to death	47 (4.75–230.75)

**Table 2:** Differences between survivors and nonsurvivors within 100 days.

Variable	Value, *n* (%)		*p* value
	Patient survived	Patient deceased	
Gender (male)	9 (81.8%)	10 (100%)	0.262
Age	42.0 ± 10.3	44.5 ± 14.8	0.572
DM	2 (18.2%)	4 (40.0%)	0.268
Coinfection			
CMV	4 (36.4%%)	3 (30.0%)	0.562
Bacteria	5 (45.5%)	5 (50.0%)	0.590
Leukemia relapse	3 (27.3%)	1 (10.0%)	0.331
GVHD	2 (18.2%)	6 (60.0%)	0.063
Hemoptysis	4 (36.4%)	9 (90.0%)	0.017^a^
WBC	2.4 (1.6–3.2)	1.3 (0.1–3.3)	0.307
NE	0.9 (0.4–1.9)	0.7 (0.08–1.4)	0.647
CRP	196.5 ± 92.5	255.7 ± 115.3	0.291
Multilobar	8 (72.7%)	9 (100%)	0.145
Cavitary infiltrate	6 (54.5%)	4 (44.4%)	0.500
Reversed halo sign	3 (27.3%)	3 (33.3%)	0.574
Pleural effusion	5 (45.5%)	5 (55.6%)	0.500
Pneumothorax	1 (9.1%)	0 (0.0%)	0.550
*Rhizopus*	7 (77.8%)	5 (50.0%)	0.220
Extrapulmonary involvement^b^	4 (36.4%)	2 (20.0%)	0.367
Surgery	2 (18.2%)	0 (0%)	0.262

^a^ *p* value is statistically significant. ^b^When the observation time was extended to 365 days, no extrapulmonary involvement occurred in the survival group and all patients with extrapulmonary involvement died within 1 year. CMV: cytomegalovirus; NE: neutrophils.

## Data Availability

The data used to support the findings of this study are available from the corresponding author upon request.

## Supplementary Materials

Supplementary Materials
*Supplementary Table 1.* Demographic characteristics of studied patients with hematological malignancies complicated with pulmonary mucormycosis.
