# Supplementary material for: Clinical Manifestations of Pulmonary Mucormycosis in Recipients of Allogeneic Hematopoietic Stem Cell Transplantation: A 21-Case Series Report and Literature Review
Source: Can Respir J. 2022 Jun 2;2022:1237125. doi: 10.1155/2022/1237125 (PMC9184213; doi:10.1155/2022/1237125)
Supplement: Supplementary Materials — Supplementary Table 1. Demographic characteristics of studied patients with hematological malignancies complicated with pulmonary mucormycosis. [file 1237125.f1.docx]

Supplementary Table1 Demographic characteristics of studied patients with hematological malignancies complicated with pulmonary mucormycosis

| Case | Age/Sex | Haematological malignancy | Leukemia relapse | GVHD before infection | Other Clinical Conditions | Extrapulmonary Involement | Diagnose | | | Antifungal Threapy | Outcome and cause of death |
| --- | --- | --- | --- | --- | --- | --- | --- | --- | --- | --- | --- |
|  |  |  |  |  |  |  | Specimen Type | Culture/ Pathology | Pathogen (Genera) |  |  |
| 1 | 43/M | Day 90 after allo-HSCT, AA | No | No | Kpn +, PA +, CMV+ | CNS (Multiple lesions in the left cerebellum) | CT-guided percutaneous lung biopsy | +/+ | *Rhizopus* | L-AMB+POS+surgery | Expired, directly attributed to mucormycosis |
| 2 | 31/M | Day 286 after allo-HSCT, AML | No | No | None | No | CT-guided percutaneous lung biopsy | +/+ | *Rhizopus* | L-AMB +POS | cured, follow-up for 1630 days |
| 3 | 40/F | Day 114 after allo-HSCT, AML | Hematologic relapse | No | None | CNS ( Right basal ganglia) | CT-guided percutaneous lung biopsy | +/+ | *Rhizopus* | L-AMB +POS | Expired, death attributed to leukemia recurs and mucormycosis |
| 4 | 27/M | Day 73 after allo-HSCT, AML | No | No | CMV+ | CNS(Abscess at the left basal ganglia，sinus and left maxillary sinus effusion) | CT-guided percutaneous lung biopsy | -/+ | N/A | L-AMB | Expired, directly attributed to mucormycosis, cerebral hernia |
| 5 | 37/M | Day 5 after allo-HSCT, AML | No | aGVHD (skin) | None | CNS (Multiple lesions in bilateral cerebellum, occipital and frontal lobes,basal ganglia, right temporal lobe and parietal cortex) | TBLB | +/- | *mucor* | L-AMB+POS | Expired, directly attributed to mucormycosis, hemoptysis |
| 6 | 46/M | Day 136 after allo-HSCT, AML | No | No | HRSV+ | No | Airway biopsy by tracheoscopy | +/+ | *Rhizopus* | L-AMB+POS | Expired, directly attributed to mucormycosis |
| 7 | 46/F | Day 11 after allo-HSCT, AML | No | No | None | No | CT-guided percutaneous lung biopsy | -/+ | N/A | L-AMB+POS+surgery | cured, follow-up for 1793 days |
| 8 | 42/M | Day 40 after allo-HSCT, ALL | No | no | CMV+ | No | CT-guided percutaneous lung biopsy | +/+ | *Rhizopus* | L-AMB for once | Expired, directly attributed to mucormycosis, MOF |
| 9 | 13/M | Day 1 after allo-HSCT, ALL | No | aGVHD (gastro-intestinal tract) | None | CNS (Right basal ganglia) | pleural effusion | +/- | *Rhizopus* | died before diagnose | Expired, directly attributed to mucormycosis |
| 10 | 54/M | Day 96 after allo-HSCT, AML | No | no | G-bacterial sepsis, Kpn+, PA+, BO , steroid-induced DM | No | sputum | +/- | *mucor* | L-AMB | cured,f ollow-up for 781 days |
| 11 | 38/M | Day 867 after allo-HSCT, ALL | No | aGVHD (skin) | CMV+，DM，Central nervous system demyelinating disease | No | sputum，peripheral blood culture | +/- | *mucor* | L-AMB | Expired, directly attributed to mucormycosis |
| 12 | 29/M | Day 764 after allo-HSCT, AML | Molecular relapse | aGVHD (skin, liver) | CMV+ ，PA+ | No | sputum | +/- | *Rhizopus* | L-AMB | cured, follow-up for 117 days |
| 13 | 54/M | Day 61 after allo-HSCT, MDS | No | No | CMV+，PA+ | No | sputum | +/- | *Rhizopus* | POS | cured, follow-up for 696 days |
| 14 | 40/M | Day 3 after allo-HSCT, MDS | No | No | Kpn,+ S. maltophilia+ | No | sputum | +/- | *Rhizopus* | L-AMB+POS | Expired, directly attributed to mucormycosis, DAH |
| 15 | 55/M | Day 43 after allo-HSCT, MDS | No | No | steroid-induced DM | No | sputum | +/- | *Rhizopus* | POS | Expired, death from progression of primary pathogenesis |
| 16 | 49/M | Day 163 after allo-HSCT, CML | Molecular relapse | No | S. epidermidis sepsis | No | sputum | +/- | *Rhizopus* | L-AMB+POS | cured, follow-up for 1129 days |
| 17 | 40/M | Day 1924 after allo-HSCT, AML | Hematologic relapse | No | None | No | sputum | +/- | *mucor* | L-AMB for once | Expired, directly attributed to mucormycosis, MOF |
| 18 | 56/M | Day 787 after allo-HSCT, AML | No | cGVHD | PA+，DM | skin and soft tissue(Left thumb，right lower eyelid) | sputum | +/- | *mucor* | died before diagnose | Expired, directly attributed to mucormycosis, MOF |
| 19 | 67/M | Day 97 after allo-HSCT, AML | No | aGVHD (gastro-intestinal tract) | steroid-induced DM, E. faecium sepsis | No | sputum | +/- | *Rhizopus* | died before diagnose | Expired, directly attributed to mucormycosis, MOF |
| 20 | 43/M | Day 172 after allo-HSCT, AML | No | aGVHD (gastro-intestinal tract) | HRSV+, DM | No | sputum | +/- | *mucor* | L-AMB | Expired, directly attributed to mucormycosis, DAH |
| 21 | 60/M | Day 172 after allo-HSCT, MDS | No | No | Kpn sepsis | No | Peripheral blood for NGS and TBLB for culture and histology | -/+ | *Rhizomucor* | L-AMB+POS | Expired, directly attributed to mucormycosis, MOF |

# L-AMB liposomal amphotericin *B,* POS posaconazole, MOF multi-organ failure, DAH [diffuse](link:diffuse) [alveolar](link:alveolar) [hemorrhage](link:hemorrhage),TBLB transbronchial lung biopsy, AA aplastic anemia, Kpn Klebsiella pneumoniae, PA Pseudomonas Aeruginosa, E.faecium Enterococcus faecium , HRSV Human respiratory syncytial virus

# CMV Cytomegalovirus, S. maltophilia Stenotrophomonas maltophilia，S. epidermidis Staphylococcus epidermidis, BO bronchiole obliterans，AML acute myeloblastic leukemia, ALL Acute lymphoblastic leukemia, MDS myelodysplastic syndrome, CML Chronic myelogenous leukemia, GVHD graft-versus-host disease, aGVHD acute graft-versus-host disease, cGVHD chronic graft-versus-host disease, allo-HSCT allogeneic hematopoietic stem cell transplantation, CNS Central nervous system, DM diabetes mellitus, NGS Next Generation Sequencing, N/A not applicable
